# RETRACTION: The role of biofield energy treatment on psychological symptoms, mental health disorders, and stress‐related quality of life in adult subjects: A randomized controlled clinical trial

**DOI:** 10.1002/jgf2.773

**Published:** 2025-01-24

## Abstract

**RETRACTION**: M. K. Trivedi, A. Branton, D. Trivedi, S. Mondal, and S. Jana, “The Role of Biofield Energy Treatment on Psychological Symptoms, Mental Health Disorders, and Stress‐Related Quality of Life in Adult Subjects: A Randomized Controlled Clinical Trial,” *Journal of General and Family Medicine* 24, no. 3 (2023): 154–163, https://doi.org/10.1002/jgf2.606.

The above article, published online on 28 January 2023 in Wiley Online Library (wileyonlinelibrary.com), has been retracted by agreement between the journal Editor‐in‐Chief, Masanobu Okayama; the Japan Primary Care Association; and John Wiley & Sons Australia, Ltd. The retraction has been agreed upon following an investigation into concerns raised by a third party, which revealed an inappropriate control group used as the placebo group of the trial, inconsistencies in the Psychological Questionnaire Scoring, highly implausible functional biomarker values that are out of the typical physiological range, and unsupported claims regarding the scientific evidence behind the biofield energy treatment. The authors were informed, however, the explanation and the partial raw data provided were deemed insufficient to address the concerns. Thus, the editors have lost confidence in the presented data and consider the results and conclusions of this manuscript insufficiently supported and substantially compromised. The authors disagree with the retraction.

**RETRACTION**: M. K. Trivedi, A. Branton, D. Trivedi, S. Mondal, and S. Jana, “The Role of Biofield Energy Treatment on Psychological Symptoms, Mental Health Disorders, and Stress‐Related Quality of Life in Adult Subjects: A Randomized Controlled Clinical Trial,” *Journal of General and Family Medicine* 24, no. 3 (2023): 154–163, https://doi.org/10.1002/jgf2.606.

The above article, published online on 28 January 2023 in Wiley Online Library (wileyonlinelibrary.com), has been retracted by agreement between the journal Editor‐in‐Chief, Masanobu Okayama; the Japan Primary Care Association; and John Wiley & Sons Australia, Ltd. The retraction has been agreed upon following an investigation into concerns raised by a third party, which revealed an inappropriate control group used as the placebo group of the trial, inconsistencies in the Psychological Questionnaire Scoring, highly implausible functional biomarker values that are out of the typical physiological range, and unsupported claims regarding the scientific evidence behind the biofield energy treatment. The authors were informed, however, the explanation and the partial raw data provided were deemed insufficient to address the concerns. Thus, the editors have lost confidence in the presented data and consider the results and conclusions of this manuscript insufficiently supported and substantially compromised. The authors disagree with the retraction.

